# Pancreatic functions in high salt fed female rats

**DOI:** 10.14814/phy2.12443

**Published:** 2015-07-26

**Authors:** Noha N Lasheen

**Affiliations:** Physiology Department, Faculty of Medicine, Ain Shams UniversityCairo, Egypt

**Keywords:** Amylase, high salt, IL6, insulin, lipase, TGF-*β*1

## Abstract

Salt consumption has been increased worldwide and the association of high salt diets with enhanced inflammation and target organ damage was reported. Little data were available about the effect of high salt diet on exocrine function of pancreas, while the relation between high salt intake and insulin sensitivity was controversial. This study was designed to investigate the effect of high salt diet on exocrine and endocrine pancreatic functions, and to elucidate the possible underlying mechanism(s). Twenty adult female Wistar rats were randomly divided into two groups; control group; fed standard rodent diet containing 0.3% NaCl, and high salt fed group; fed 8% NaCl for 8 weeks. On the day of sacrifice, rats were anesthized by i.p. pentobarbitone (40 *μ*g/kg B.W.). Nasoanal length was measured and fasting blood glucose was determined from rat tail. Blood samples were obtained from abdominal aorta for determination of plasma sodium, potassium, amylase, lipase, aldosterone, insulin, transforming growth factor-*β* (TGF-*β*1), and interleukin 6 (IL6). Pancreata of both groups were histologically studied. Compared to control group, 8-week high salt fed group showed: significant elevation in body weight, body mass index, Lee index, plasma sodium, TGF-*β*1 and IL6, however, plasma aldosterone, amylase, lipase, and insulin levels were significantly decreased. A nonsignificant increase in plasma potassium and nonsignificant changes in fasting blood glucose and HOMA-IR were detected between groups. Pancreatic fibrosis was observed in test group. High salt diet for 8 weeks caused pancreatic fibrosis evidenced by decline of both exocrine and endocrine functions of pancreas in Wistar rats.

## Introduction

The consumption of certain nutritional diets such as fructose, fat, and salt has increased in recent years throughout the world (Madero et al. 2011). Experimental and clinical research has shown that the increased levels of these components in processed food were related to high body mass, hypertension and insulin resistance. Recent studies suggested that diets high in sodium salts were associated with enhanced inflammation, lipid disorders, target organ damage, and also with hypertension and decline in renal function (Ohta et al. 2013). In humans and animal models, it has been observed that excess salt intake impaired endothelial function, increased serum uric acid, and decreased nitric oxide production, resulting in impairment a homeostatic mechanism regulating blood pressure in the body (Forman et al. 2012). In addition, excessive salt intake has been reported to decrease insulin sensitivity (Baudrand et al. 2014).

The pathogenic effects of high salt were reported to be elevating blood pressure (Messerli et al. 1997), tissue injury, and promoting organ hypertrophy such as myocardial interstitial collagen (Conrad et al. 1995). Moreover, high salt was reported to be a mediator of tissue fibrosis despite aldosterone infusion (Brilla and Weber 1992).

Aldosterone normally caused sodium reabsorption in the distal nephron via the mineralocorticoid receptor; in addition to extrarenal effects. It could induce myocardial fibrosis and vascular injury without severe hypertension (Rocha et al. 2000). Aldosterone excess produced insulin resistance in animals and humans (Colussi et al. 2007), could be progressed to overt type 2 diabetes mellitus (Kahn et al. 2006). In addition, Mosso et al. (2007) observed an inverse relation between insulin secretion and aldosterone levels in hypertensive patients. Furthermore, blockade of the renin–angiotensinogen–aldosterone system has beneficial effects on glucose homeostasis through unknown mechanism (McMurray et al. 2010).

However, Luther (2011) demonstrated that aldosterone deficiency or excess modulated insulin secretion in vivo and in vitro via reactive oxygen species and in an independent manner of mineralocorticoid receptors.

Transforming growth factor-*β* (TGF-*β*), a three growth factors family, plays a role on organ development and cell growth, and could have major importance in the expression of extracellular matrix proteins and promotion of vascular and renal fibrosis in many disease states (Border et al. 1990). TGF-*β*1 is considered the most important mammalian TGF-*β* family member (Kanbay et al. 2011).

Cytokines, especially TGF-*β*1, could play a role in promoting fibrosis such as the kidney and blood vessels (Border and Noble 1994). Overexpression of TGF-*β*1 was observed in many models of progressive renal injury and cardiovascular disease (Lorrell 1997; Weber 1997), who stated that TGF-*β*1 played a role in dietary salt-induced organ hypertrophy and fibrosis, and investigated the salt intake effects including tissue fibrosis that could cause organ failure.

Ying and Sanders (1998) showed that high salt intake in normotensive rats rapidly increased endothelial active TGF-*β* production. Moreover, Yu et al. (1998) showed fibrosis of the hearts and kidneys in rats fed 8% salt diet for 8 weeks. In addition, interleukin 6 (IL-6) was reported to be a profibrogenic cytokine. It has been shown to increase fibroblast collagen (Spörri et al. 1999).

Based on the previous studies, it could be observed that a high salt diet could induce insulin resistance and could produce insulin secretion. Exocrine function of pancreas was not widely investigated in high salt diet state. Therefore, it is of great clinical interest to elucidate the effects and the underlying mechanism(s) of high salt diet effects on different organs such as pancreas.

### Aim of the work

The present study aimed at investigating the effects of high salt diet for 8 weeks on both endocrine and exocrine pancreatic functions in adult female rats, and to elucidate the possible underlying mechanism(s) of such effects.

### Site of research

Physiology Department, Faculty of Medicine, Ain Shams University, Cairo, Egypt.

## Materials and Methods

### Experimental protocol

#### Animals

Animals used were 20 adult female Wistar rats, initially weighing 130–150 g. Rats were purchased from the Research Institute of Ophthalmology, Giza, Egypt, and housed in animal cages (four rats/cage) with suitable ventilation, temperature of 22–25°C, 12-h light–dark cycle and free access to food and water – ad libitum – in the Animal House, Physiology Department, Faculty Of Medicine, Ain-Shams University.

They were allowed to the new environment for 7 days prior to experimental procedures to decrease the possible discomfort of animals.

Animals were randomly allocated into the following groups:

Group-I: Control group (*n* = 10): They received a regular diet composed of bread, milk, and Karish cheese with free access to water. They were fed standard rodent diet containing 0.3% NaCl (Liu et al. 2010).
Group-II: 8 week High salt Diet Fed group (*n* = 10): They were fed a diet containing 8% NaCl (Liu et al. 2010) for 8 weeks (Yu et al. 1998).

#### Preparation of dietary formula

The preparation of control and high salt diet was achieved by calculations which depend upon the analytical composition of different food sources used in animal feeding in the laboratory.

#### Preparation of control diet

The diet was prepared in Animal House laboratory from three main natural sources, bread (Balady bread), pasteurized cow milk, and unsalted Karish cheese. Every 100 g of control diet was prepared by mixing 75 g bread, 123 mL milk (equivalent to 15 g dry weight), and 10 g unsalted Karish cheese.

#### Preparation of high salt diet

The high salt chow was prepared by mixing 76 g of NaCl with 924 g of chow (Sofola et al. 2002).

The rats in both groups were fed on their corresponding diets for 8 weeks with water given ad libitum.

At the end of experimental period, overnight-fasted rats were anesthetized with intraperitoneal injection of Pentobarbital (40 mg/kg B.W.). All rats were subjected to the following experimental studies:

- Measurement of body weight (BW) and naso-anal length for calculations of some obesity indices:
- Determination of body mass index (BMI): It was calculated according to Bernardis (1970)*,* as follows:

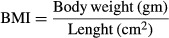

- Determination of Lee index: was calculated according to Bernardis (1970) as follows: 

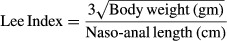

- A blood drop from the tail was taken for immediate blood glucose measurement by the GlucoDr™ SuperSensor Test Meter, AGM- 2200, Korea.
- An abdominal midline incision was performed, collection of blood samples from the abdominal aorta was performed, then centrifuged to separate the plasma which was used for subsequent determination of sodium, potassium, aldosterone, insulin, amylase, and lipase levels colorimeterically using commercially available kits, also TGF-*β*1 and Interleukin 6 levels were determined using ELISA kits. Samples of pancreatic tissues were fixed in 10% formalin for light microscopy to be histologically studied.

The homoeostasis model assessment-insulin resistance (HOMA-IR) index was calculated from fasting plasma glucose and insulin concentrations according to Bonora et al. (2000) equation: insulin (in mU/L) × glucose (in mmol/L)/22.5

### Statistical analysis

All results in the current study were expressed as mean ± SE of the mean, using Statistical Package for the Social Sciences (SPSS, Inc., Chicago, IL) program, version 20.0 to compare significance between the two groups. The comparisons were made using unpaired “*t*” test. Differences were considered significant when *P* ≤ 0.05.

### Ethics committee

This study was approved by the Ethics Committee of Faculty of Medicine, Ain Shams University.

## Results

**Figure 1:**
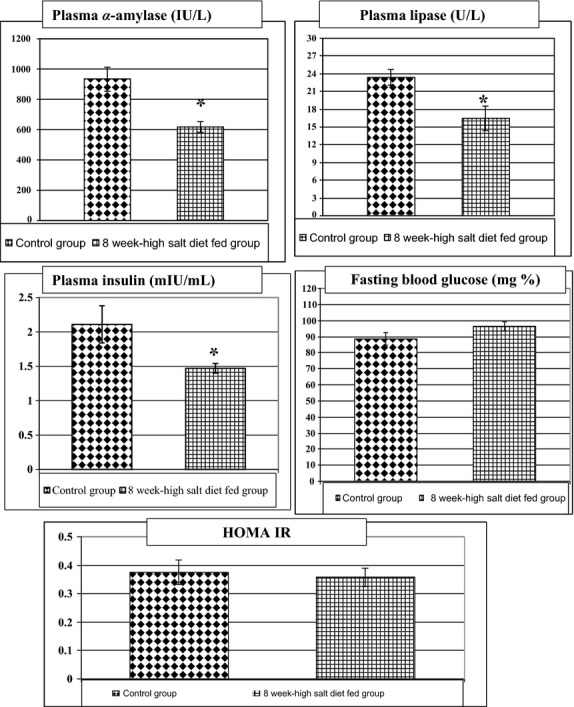
Exocrine and endocrine functions of pancreas in the different studied groups. *Significance by LSD at *P* < 0.05 from control group.

Regarding the exocrine function of pancreas, Table1 and Figure1 show that 8-week high salt diet resulted in a significant decrease in both the plasma *α*-amylase (617.83 ± 36.29) and lipase levels (16.5 ± 2.08) compared to control group (934.01 ± 80.4, *P* < 0.02 & 23.38 ± 1.38, *P* < 0.02, respectively).

**Table 1:** Exocrine function of pancreas in the different studied groups

	Control group (*n* = 10)	8-week high salt diet Fed group (*n* = 10)
Plasma *α*-Amylase (IU/L)	934.01 ± 80.4	617.83 ± 36.29
*P*		<0.02
Plasma Lipase (U/L)	23.38 ± 1.38	16.5 ± 2.08
*P*		<0.02

*P*: Significance by LSD at *P* < 0.05 from control group.

As shown in Table2 and Figure1, plasma insulin as a marker of pancreatic endocrine function, was significantly decreased in 8-week high salt diet fed rats compared to control rats (1.47 ± 0.07 vs. 2.11 ± 0.27, *P* < 0.05). However, fasting blood sugar (96.5 ± 2.67 vs. 88.5 ± 3.91) and HOMA-IR (0.357 ± 0.032 vs. 0.375 ± 0.044) showed nonsignificant changes between the two groups.

**Table 2:** Endocrine function of pancreas in the different studied groups

	Control group (*n* = 10)	8-week high salt diet Fed group (*n* = 10)
Plasma insulin (mIu/mL)	2.11 ± 0.27	1.47 ± 0.07
*P*		<0.05
Fasting blood sugar (mg%)	88.5 ± 3.91	96.5 ± 2.67
*P*		NS
HOMA-IR	0.375 ± 0 .044	0.357 ± 0.032
*P*		NS

*P*: Significance by LSD at *P* < 0.05 from control group.

**Figure 2:**
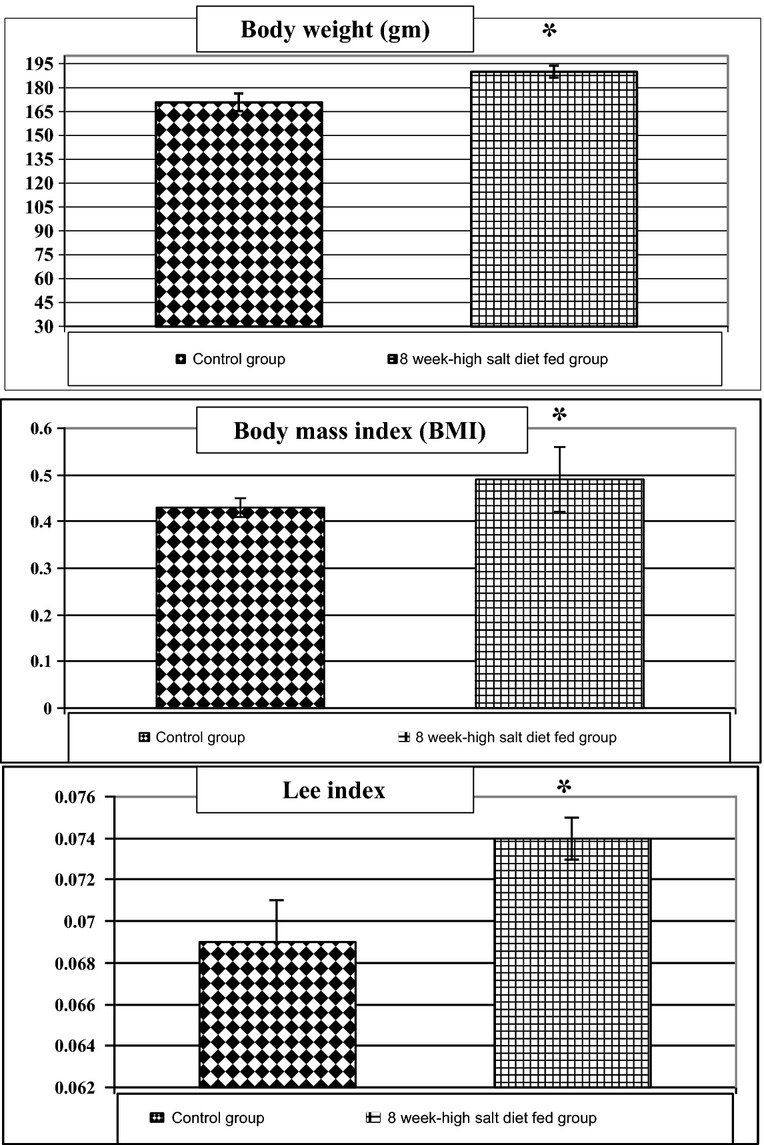
Changes in body weight (mg), body mass index (BMI), and Lee index in the different studied groups. *Significance by LSD at *P* < 0.05 from control group.

On the other hand, compared to control group, 8-week high salt diet fed group showed a significant increase in body weight (190 ± 3.78 vs. 170.83 ± 5.54, *P* < 0.02), body mass index (BMI) (0.49 ± 0.07 vs. 0.43 ± 0.02, *P* < 0.05), and Lee index (0.074 ± 0.001 vs. 0.069 ± 0.002, *P* < 0.05) as shown in Table3 and Figure2.

**Table 3:** Changes in body weight (gm), Body mass index (BMI), and Lee index in the different studied groups

	Control group (*n* = 10)	8-week high salt diet Fed group (*n* = 10)
Body Weight (gm)	170.83 ± 5.54	190 ± 3.78
*P*		<0.02
BMI	0.43 ± 0.02	0.49 ± 0.07
*P*		<0.05
Lee Index	0.069 ± 0.002	0.074 ± 0.001
*P*		<0.05

*P*: Significance by LSD at *P* < 0.05 from control group.

**Figure 3:**
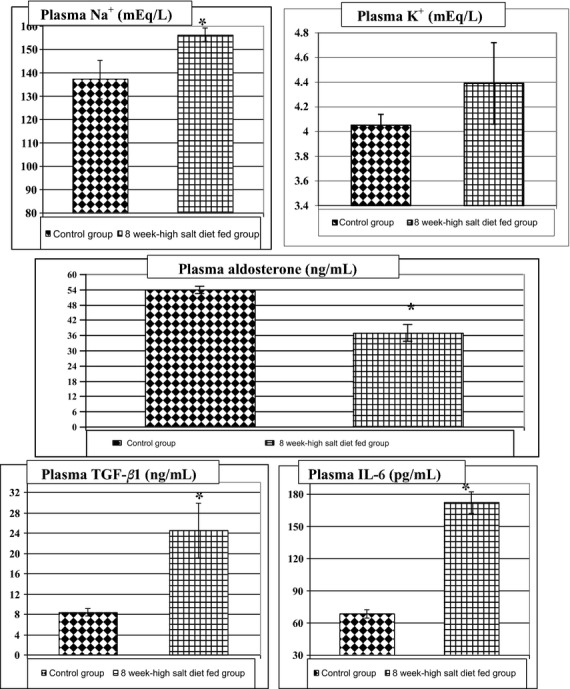
Plasma levels of sodium (mEq/L), potassium (mEq/L), aldosterone (ng/mL), TGF-β1 (ng/mL), and IL-6 (pg/mL) in the different studied groups. *Significance by LSD at *P* < 0.05 from control group.

As shown in Table4 and Figure3, 8-week high salt diet fed group showed a significant increase in plasma sodium level (156.27 ± 2.8 vs. 137.16 ± 8.14, *P* < 0.05) accompanied by a significant decrease in plasma aldosterone level (37.04 ± 3.23 vs. 53.97 ± 1.46, *P* < 0.005) compared to control group. However, plasma potassium level showed insignificant rise in the 8-week high salt diet fed group compared to the control group (4.39 ± 0.33 vs. 4.05 ± 0.09).

**Table 4:** Changes in plasma levels of sodium (mEq/L), potassium (mEq/L), and aldosterone (ng/mL) in the different studied groups

	Control group (*n* = 10)	8-week high salt diet Fed group (*n* = 10)
Plasma Na^+^ (mEq/L)	137.16 ± 8.14	156.27 ± 2.8
*P*		<0.05
Plasma K^+^ (mEq/L)	4.05 ± 0.09	4.39 ± 0.33
*P*		NS
Plasma Aldosterone (ng/mL)	53.97 ± 1.46	37.04 ± 3.23
*P*		<0.005

*P*: Significance by LSD at *P* < 0.05 from control group.

In addition, plasma TGF-*β*1 and IL6 were significantly elevated in 8-week high salt fed group compared to control group (24.56 ± 5.4 vs. 8.41 ± 0.7, *P* < 0.02; 172.37 ± 9.91 vs. 68.44 ± 3.64, *P* < 0.001, respectively), as shown in Table5 and Figure3.

**Table 5:** Changes in TGF- B1 (ng/mL) and IL6 (pg/mL) in the different studied groups

	Control group (*n* = 10)	8-week high salt diet Fed group (*n* = 10)
Plasma TGF-*β*1 (ng/mL)	8.41 ± 0.7	24.56 ± 5.4
*P*		<0.02
Plasma IL6 (pg/mL)	68.44 ± 3.64	172.37 ± 9.91
*P*		<0.001

*P*: Significance by LSD at *P* < 0.05 from control group.

### Histological results

**Figure 4:**
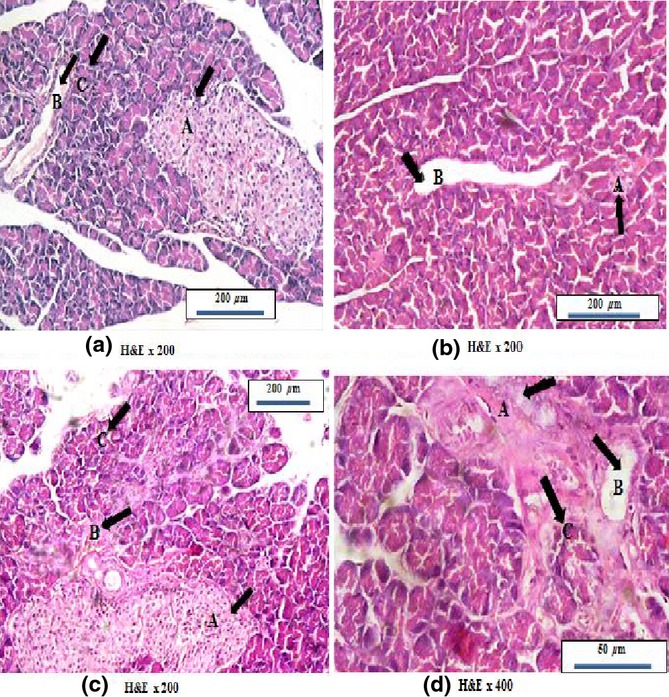
Photomicrographs of rat pancreas stained with H&E; (a) Control pancreas showing normal acinar arrangement with basal basophilia and apical acidophlia (black arrow &C) and normal sized interlobular duct (black arrow &B), with normal sized islet of Langerhans (black arrow &A). (b) Pancreas of high salt diet fed group showing widened interlobular duct (black arrow &B) and degenerated islet of Langerhans (black arrow &A). (c) Pancreas of high salt diet fed group showing degenerated acini (black arrow &C) with burden interlobular duct (black arrow &B) inside C.T septa in addition to fused islets of Langerhans (black arrow &A). (d) Pancreas of high salt diet fed group showing expanded fibrous tissue septa with disruption of acinar arrangement appeared as entrapped acini (black arrow &C) with widened interlobular duct (black arrow &B) and also degenerated entrapped islet of Langerhans (black arrow &A).

As shown in Figure4, rat pancreas of control group showed normal arrangement of acini with basal basophilia and apical acidophilia with small-sized interlobular duct contains homogenous pink-staining protein-rich pancreatic juice, as well as normal configuration of islets of Langerhans (4a, H&E X200).

Pancreas of 8-week high salt diet fed group showed distortion of normal acinar lobular arrangement which appeared deeply basophilic with pyknotic nuclei and less acidophilic secretion and widened interlobular duct (4b&c, H&E x200), also, islet of Langerhans was degenerated and atrophied (4b, H&E x200). In addition, pancreas of 8-week high salt diet fed group showed expanded fibrous tissue into the lobules affecting acinar arrangement and causing fusion of islets of Langerhans (4c, H&E x400). Moreover, there were some acini entrapped inside the newly formed fibrous tissues together with distorted islets of Langerhans (4d, H&E x400).

**Figure 5:**
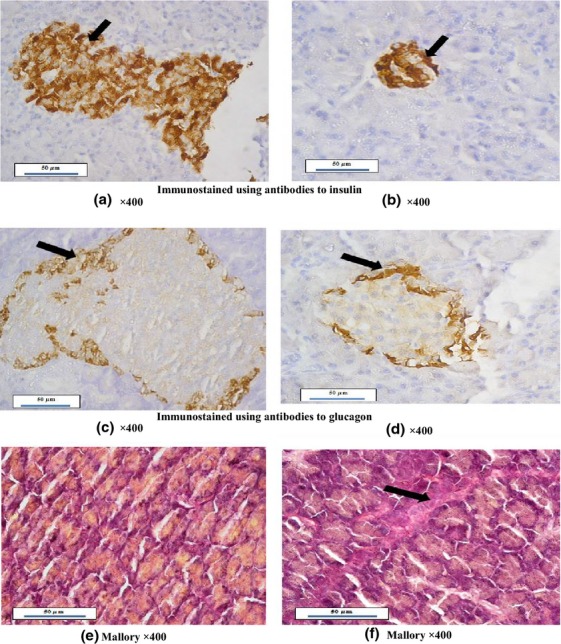
Photomicrographs of rat pancreas; (a) Control pancreas immunostained with antibodies to insulin showing normal sized islet of Langerhans with dense β cells (black arrow). (b) Pancreas of high salt diet fed group immunostained with antibodies to insulin showing degenerated islet of Langerhans with less positive β cells (black arrow). (c) Control pancreas immunostained with antibodies to glucagon showing α cells surrounding the islets of Langerhans (black arrow). (d) Pancreas of high salt diet fed group immunostained with antibodies to glucagon showing degenerated islets of Langerhans with less α cells reaction located at the islet border (black arrow). (e) Control pancreas stained with Mallory stain showing normal arrangement of acini filled with secretions without fibrosis. (f) Pancreas of high salt diet fed group in Mallory stain showing dense fibrous tissues distorting the acini (black arrow).

As shown in Figure5, immunohistochemical identification of insulin and glucagon revealed that 8-week high salt diet resulted in less positive reaction and decreased *β* cell size in islets of Langerhans (5b X400) compared to control group (5a X400), also, *α* cell size in degenerated islets of Langerhans was decreased and showed less positive staining in 8-week high salt diet fed group (5d X400) compared to control group (5c X400).

Pancreatic histopathological examination with Mallory staining showed that 8-week high salt diet fed group caused increased fibrous tissue distorting acinar arrangement (5f X400) compared to control group (5e X400) that had normal acinar arrangement.

## Discussion

The current study showed that high salt diet for 8 weeks caused significant decrease in plasma levels of *α*-amylase and lipase, a significant decrease in plasma insulin, a significant increase in body weight, BMI, and Lee index accompanied by a significant increase in plasma sodium, TGF-*β*1, and IL6, and a significant decrease in plasma aldosterone compared to the control group. Meanwhile, HOMA-IR and fasting blood glucose were insignificantly different, and plasma potassium was increased nonsignificantly in the 8-week high salt fed group compared to the control group.

The decline in exocrine pancreatic function, proved by the significant decrease in plasma levels of *α*-amylase and lipase in the 8-week high salt fed group, indicates a salt-induced impairment of exocrine pancreatic function. This could be due to fibrotic changes in pancreatic tissue induced by high salt. The significantly elevated plasma TGF-*β*1and IL6 found in 8-week high salt fed group supports this concept. This finding was in agreement with Lorrell (1997) and Weber (1997), who found that salt intake could cause tissue fibrosis, and subsequent organ failure. Thus, this work is a novel study demonstrating the effect of high salt diet on exocrine pancreatic function.

Pancreatic fibrosis usually resulted from repeated manifest or silent attacks of acute pancreatitis, causing the permanent pancreatic destruction leading to pancreatic exocrine insufficiency and/or endocrine failure (Andersen 2007). In advanced chronic pancreatitis, the acinar tissue might be so atrophic that there is no source for pancreatic enzymes (Longnecker 1982), resulting in pancreatic exocrine insufficiency. Clinically, measurement of exocrine function is assessed by either direct or indirect pancreatic function tests. Direct function tests depend on measurement of pancreatic enzymes in the juice collected via a duodenal tube, and might be the gold standard for assessing exocrine pancreatic function (Aghdassi et al. 2011). Indirect function tests could detect decreased pancreatic enzymes in stool or serum or, alternatively, assess the digestion of administered orally synthetic substrates (Keller et al. 2009).

The high salt-induced fibrosis, evidenced by elevation of plasma TGF- *β*1 and distorted acinar arrangement observed in Mallory stain examination, obtained in the present study, was in alignment with the study of Yu et al. (1998). They stated that high dietary salt led to widespread fibrosis and increased TGF-*β*1 in the heart and kidney in normotensive and hypertensive rats. They suggested a specific effect of dietary salt on fibrosis, possibly via TGF-*β*1-dependent pathways, and also, suggested that excessive salt intake could be an important direct pathogenic factor for cardiovascular disease.

The increased interleukin-6 (IL6) and TGF-*β*1, obtained in the present study, in 8-week high salt fed group is strongly supported by the advanced fibrotic histological appearance of the exocrine and the endocrine parts of the pancreas.

It was stated that increased expression of TGFs could play an important role in pancreatic repair and remodeling after acute edematous pancreatitis. The increased level of one or more members of TGF family might contribute to the changes in the extracellular matrix and to the repair of the pancreatic acinar and ductal cells. Increased expression of transforming growth factors after acute edematous pancreatitis in rats suggests a role in pancreatic repair (Riesle et al. 1997).

In addition, fibrosis could strongly depend on IL6. Repeat inflammation caused IL6-mediated T helper 1 (Th1) cell effector commitment and the emergence of signal transducer and activator of transcription-1 (STAT1) activity within the peritoneal membrane. IL6 could compromise tissue repair by shifting acute inflammation into a more chronic profibrotic state by stimulating Th1 cell responses as a consequence of recurrent inflammation (Fielding et al. 2014). Thus, the significant rise of IL6 found in the 8-week high salt fed group could explain the pancreatic damage observed histopathologically, in the form of fibrosis and acinar distortion, and functional impairment evidenced by decreased plasma levels of amylase and lipase. Sathyanarayan et al. (2007) reported that increased IL6 levels could predict organ failure and severe pancreatitis, and they suggested its pathophysiological significance in acute pancreatitis.

TGF-*β*_1_, as a major profibrogenic cytokine, plays a role in pancreatic fibrosis. Also, IL6, a proinflammatory cytokine, could participate in pancreatic fibrosis by activating pancreatic stellate cells (PSCs). Moreover, there is autocrine interplay between these two primary factors responsible for pancreatic fibrosis. TGF-*β*1 acts locally to increase IL-6 secretion of activated PSCs (Aoki et al. 2006a).

The salt-induced fibrosis of pancreatic tissue in the 8-week high salt fed group could be further supported by the significant decrease in plasma insulin in the 8-week high salt fed group. This indicates a decline in pancreatic endocrine function in response to high salt loading. Also, the fibrotic changes observed, in the current study, in the form of acinar fibrosis with distorted arrangement and some entrapped acini inside the newly formed fibrous tissues in 8 week high salt fed group, could explain exocrine function impairment. Also, the degenerated islets of Langerhans could explain the decline in pancreatic endocrine function, that was observed by decreased beta cell size by using anti-insulin antibodies immunohistochemistry.

Nonsignificant changes were detected between both groups in plasma glucose level and HOMA-IR. The pancreatogenic diabetes, which is the endocrine dysfunction in chronic pancreatitis, could appear as abnormal glucose tolerance, and could be converted into overt diabetes mellitus due to loss of insulin secretion or insulin resistance (Aghdassi et al. 2011). Hypoglycemic episodes could occur and might be lethal due to a lack of counter control caused by the parallel absence of glucagon (Andersen 2007), that was evidenced in the current study by less positive reaction in immunohistochemistry against glucagon in 8-week high salt fed group when compared to control group. It was noted, in the present study, that intraperitoneal glucose tolerance test was performed on the day before sacrifice day, and revealed nonsignificant changes between both groups. Thus, insulin resistance could not be proved in the current study, especially due to the absence of intraperitoneal insulin tolerance test.

The nonsignificant change in plasma glucose level, found in the current study, is in accordance with Liu et al. (2010), as they found no effects on fasting glucose in the normoglycemic rat model. Moreover, Fonseca-Alaniz et al. (2008) found that long-term consumption of a high sodium diet for 9 weeks influenced glucose and insulin serum concentration in rats. They found that the high sodium intake could interfere with glucose and insulin metabolism, and that chronic salt overload enhanced adipocyte insulin sensitivity to glucose uptake and increased adipose visceral masses.

The significantly lowered plasma insulin level obtained in 8-week high salt fed group could be explained by deceased β cell content evidenced by immunohistochemical identification against insulin. This significant decrease in plasma insulin in 8-week high salt fed group disagrees with the study of Ogihara et al. (2001), who found that body weight, food intake, and fasting blood glucose level were not altered after salt loading for 2 weeks, whereas, hyperinsulinemic-euglycemic clamp method, used to examine the insulin sensitivity, in high salt fed rats showed that both 2 and 8 weeks of salt loading caused significant insulin resistance manifested by the decreased glucose infusion rate.

In addition, the significant lowered plasma insulin in 8 week high salt diet fed group, obtained in the current study, was not accompanied by high blood glucose level despite of histological findings of distortion of islets of Langerhans. That could be attributed to Yeo et al. (1989), who stated that no definitive correlation between pancreatic structure and endocrine function has been reported. Pancreatic endocrine deficiency, as measured by a reduced insulin response to glucose loading, might also be speculated to involve a combination of factors such as altered pancreaticoportal blood flow, impaired neural innervation of islet cell tissue, or impaired paracrine regulatory mechanisms involving other islet cells. Pancreatic ductal stenosis with resultant pancreatic fibrosis and chronic pancreatitis is associated with glucose intolerance and lack of appropriate islet responsiveness leading to insulin deficiency, despite histologic and ultrastructural evidence of intact islets of Langerhans (Yeo et al. 1989).

The decreased plasma insulin level in 8-week high salt fed group could be attributed to significantly elevated IL6. That could be supported by study of Sandler et al. (1990), who suggested a role for IL6 in the process of B-cell suppression and destruction during the course of insulin-dependent diabetes mellitus.

The salt concentration, difference in NaCl absorption between rat chow and liquid preparation, duration of salt loading, and the older age of starting the salt diet among the various studies might explain the controversial results of insulin levels and blood glucose level in the different studies.

In addition, the current study showed a significant decrease in plasma aldosterone and a significant elevation of plasma sodium in the 8-week high salt fed group compared to the control group. This significantly decreased plasma aldosterone level observed in 8-week high salt fed group agrees with Ferreira et al. (2010) that goes with the normal homeostatic mechanisms regulating aldosterone secretion in conditions of excess sodium. Normally, there is a biological feedback mechanism to lower aldosterone levels in the presence of a high salt intake via suppression of the circulating renin–angiotensin system, a major determinant of aldosterone production by the adrenal gland (Morizane et al. 2012). Also, these findings were in accordance to Ogihara et al. (2001), who found that plasma aldosterone was significantly lowered in the high salt fed rats than in the controls. However, Takeda et al. (2000) showed an elevated activity and tissue, not plasma, synthesis of aldosterone in normotensive rats fed a high sodium diet for 8 weeks after weaning.

Also, the significant decrease in plasma insulin and aldosterone levels in 8-week high salt fed group in the present study supported by in vivo study showed that aldosterone synthase deficiency could affect insulin secretion due to the effect of increased angiotensin II. During normal sodium intake, relative volume depletion and increased angiotensin II in aldosterone-deficient mice could decrease islet blood flow and insulin secretion (Makhanova et al. 2006).

The significant decrease in plasma insulin and aldosterone levels in 8-week high salt fed group disagrees with the study of Luther et al. (2009), who suggested that aldosterone alters insulin secretion in pancreatic islets and clonal beta cells through independent mechanism of mineralocorticoid receptor and glucocorticoid receptor, and might affect reactive oxygen species generation.

The elevated plasma sodium level in 8-week high salt fed group encountered in the present study, is in agreement with the study of Visser et al. (2009), who found that high salt diet caused elevation of plasma sodium in healthy subjects after 1 week of high salt diet consumption.

However, the increased plasma sodium in the 8-week high salt fed group disagrees with the study of Gonzalez et al. (2005), who maintained adult male rats under control or a high-salt added to the drinking water during 4 days. They found no significant differences in plasma electrolytes or blood pressure among the groups. In contrast, aldosterone levels decreased in their study. Thus, the changes in sodium and potassium levels obtained in the current study could be attributed to the longer term of high salt diet being 8 weeks and to the different route of administration of high salt.

Although plasma sodium was significantly increased in the current study, nonsignificant increase was found in plasma potassium in the 8-week high salt fed group when compared to control group. It could be due to lowered plasma aldosterone level encountered in this study.

Also, body weight, BMI, and Lee index were significantly increased in the 8-week high salt fed group that could be attributed to volume expansion as evidenced by significant sodium increase in the high salt fed group and it is, also, supported by the study of Ferreira et al. (2010), who suggested a possible small expansion in extracellular volume associated with dietary salt overload. Moreover, Visser et al. (2009) studied the correlation between BMI and plasma sodium after shift from low salt diet to high salt diet in healthy subjects. They found that a shift to high salt diet leads to a larger rise in extracellular fluid volume in healthy subjects with higher BMI, associated with sodium elevation during high salt diet, although no hypertension occurred in healthy subjects. They, also, suggested that their findings could be a potential explanation for the interaction of sodium intake and BMI on cardiovascular and renal risk. The increased body weight, BMI, and Lee index in 8-week high salt diet fed group could be due to increased organ weights. This could be supported by the study of Matavelli et al. (2007), who found that left ventricular, aortic, and left kidney masses were increased in the 6% and 8% salt-loaded rats. Also, the increased body weight, BMI, and Lee index could be attributed to fat accumulation, however, visceral fat was not evaluated in the present study.

The rats used in the present study were females only to exclude the effects of sex hormones on inflammatory mechanism that could be altered after high salt diet. Bouman et al. (2005) reported that sex hormones could affect the immune system, also, Naugler et al. (2007) stated that estrogen could protect against inflammatory disease by inhibiting IL-6 secretion from Kupffer cells. However, the biochemical mechanisms for the gender disparity are unknown (Pergola et al. 2008).

Fibrosis, is the accumulation of excessive amounts of extracellular matrix proteins in a tissue is an active dynamic process that may be reversible in its early stages. Pancreatic fibrosis is a common histopathological feature of chronic pancreatitis of all etiologies. Pancreatic fibrosis is caused by activation of pancreatic stellate cells (PSCs) that normally occur in the periacinar region of the pancreas and rest quiescent (Blaine et al. 2009), and they fulfil an important function for maintenance of synthesis and degradation of the extracellular matrix (ECM) (Apte et al. 2009). Activation of pancreatic stellate cells is the most crucial step for fibrosis. Different mechanisms can activate these cells such as ethanol and its metabolites and various cytokines such as TGF-*β*1 (Vonlaufen et al. 2007). Activated pancreatic stellate cells transform into a myofibroblast-shaped cell type that is capable of migrating easily and secreting elevated amounts of extracellular matrix proteins, especially type I, laminin, and fibronectin. Moreover, cytokines, synthesized by activated stellate cells, stimulate PSCs in an autocrine loop (Aoki et al. 2006b).

It is worth noting that the present study is the first study, up to author knowledge, to demonstrate the exocrine pancreatic function in high salt diet fed rats for 8 weeks. Fibrosis of pancreas observed in 8-week high salt diet fed group as evidenced by impairment of exocrine and endocrine pancreatic functions, and also, by histological finding of ductal and acinar fibrosis, could be due to high plasma levels of TGF-*β*1 and IL6, and not due to plasma aldosterone which was decreased significantly. That could be controversial to the study of Lal et al. (2003), which showed that mineralocorticoid receptor antagonist spironolactone might prevent the development of left ventricular hypertrophy and fibrosis induced by 8-week feeding of adult Wistar rats with an 8% NaCl diet.

Therefore, increased salt intake had deleterious effects on many organ structure and function. High salt diet induces cardiac, vascular, and renal hypertrophy and fibrosis (Yu et al. 1998), in addition to, the pancreas as studied in the present work. The underlying mechanisms responsible for salt-induced tissue fibrosis include increased expression of prosclerotic cytokines, mostly TGF-*β*1 and/or IL6. Thus, it could be recommended to decrease dietary salt intake (Yu et al. 1998).

In conclusion, high salt diet for 8 weeks caused both exocrine and endocrine pancreatic insufficiency in rats. Pancreatic functions’ impairment is explained by the salt-induced pancreatic fibrosis, mediated by the high levels of TGF-*β*1 and IL6.

Thus, it is strongly recommended that exocrine pancreatic function should be further studied, in animals, from small intestinal aspirates after consumption of high salt diet for longer periods. In addition, endocrine pancreatic function should be carefully monitored in humans consuming high salt in their diets.

## Conflict of Interest

None declared.
